# Supplementary figures and images for: Rewarding animals based on their subjective percepts is enabled by online Bayesian estimation of perceptual biases
Source: PLoS Biol. 2025 May 20;23(5):e3002764. doi: 10.1371/journal.pbio.3002764 (PMC12129325; doi:10.1371/journal.pbio.3002764)

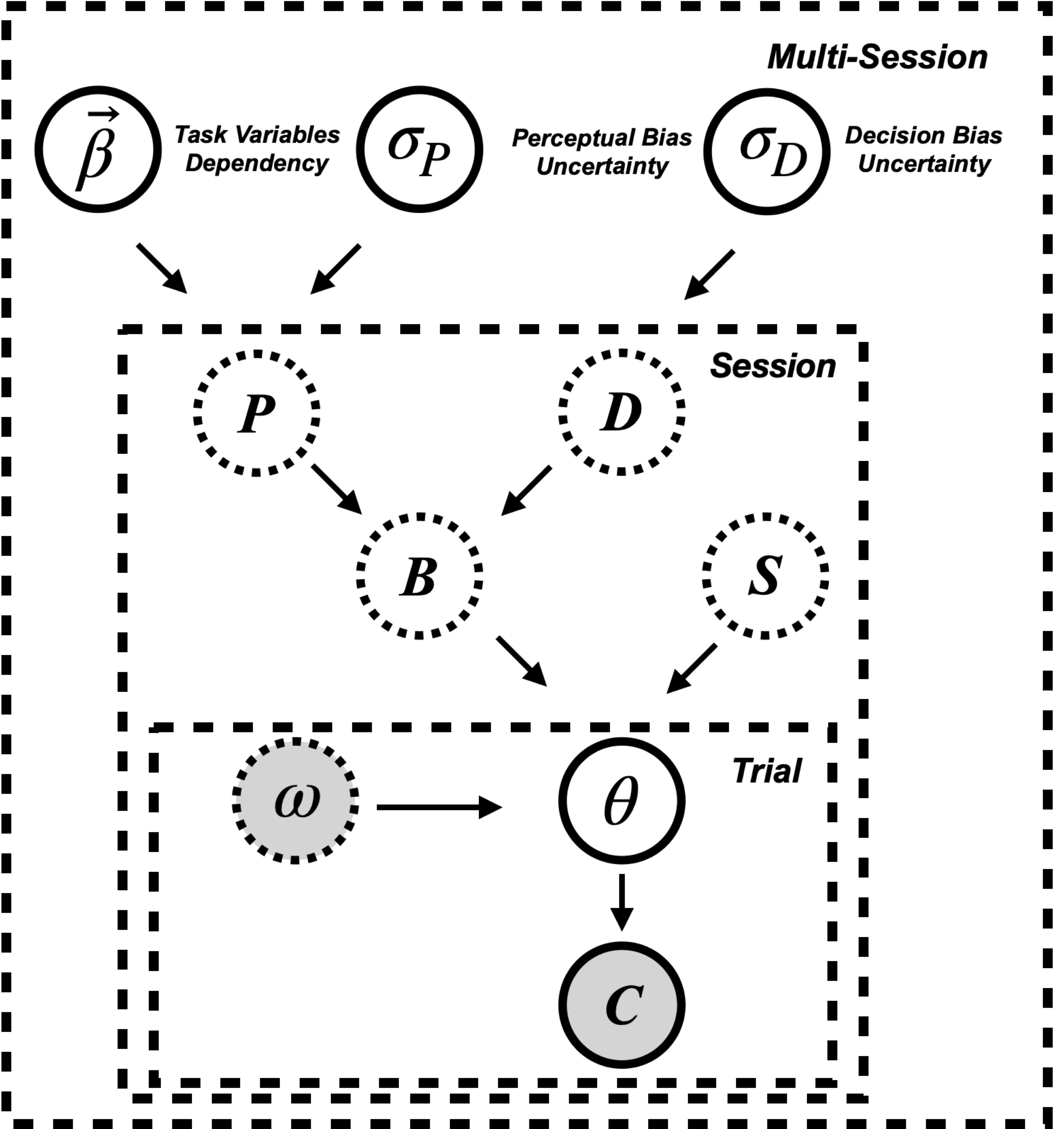

Supplement: S1 Fig — The two inner plates represent the same generative model as in Fig 4C. This model is extended by incorporating latent variables shared across sessions. The vector β→ encodes the weights that determine how perceptual biases vary with heading direction and stimulus eccentricity across sessions. σP and σD represent the standard deviations of the perceptual and decision bias random variables, respectively. See Method section “Extended hierarchical Bayesian model” for further details. (TIFF) [file pbio.3002764.s001.tif]

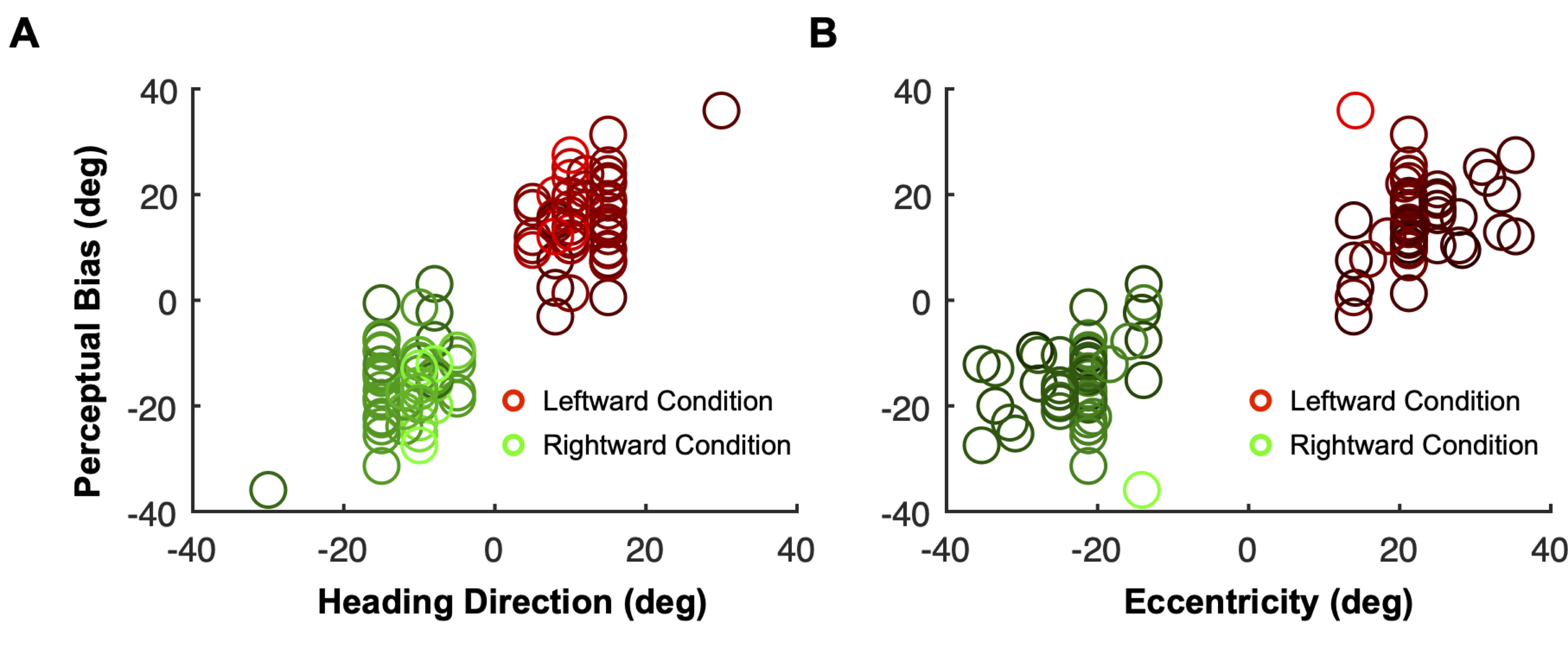

Supplement: S2 Fig — (A) Relationship between perceptual bias and the heading direction simulated by optic flow in the monkey task. With a larger heading direction, the monkey showed a larger perceptual bias in leftward and rightward conditions. Symbol colors, from light to dark, represent eccentricity, from large to small. (B) Relationship between perceptual bias and object eccentricity. With a larger eccentricity, the monkey showed a larger perceptual bias in leftward and rightward conditions. Symbol colors from light to dark represent heading directions from large to small. Source data for S2 FigA-B are available in “analysis_data/Figure7BCD&FigureS2.mat” at https://doi.org/10.5281/zenodo.15341390. (TIFF) [file pbio.3002764.s002.tif]

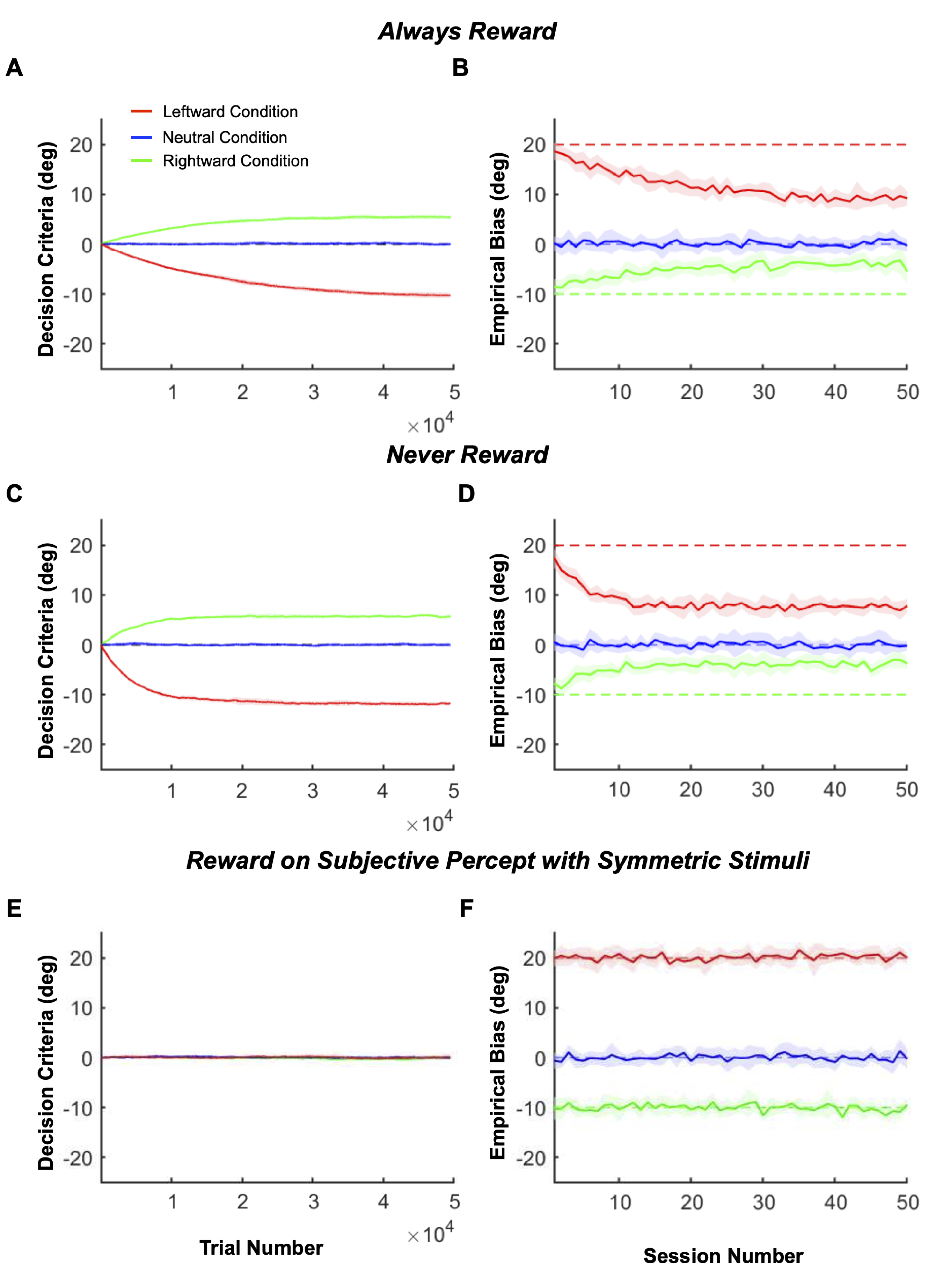

Supplement: S3 Fig — (A, B) Same as (Fig 3A, 3B), but with an “always reward animals in ambiguous trials” strategy. (C, D) Same as Fig 3A, 3B, but simulating a “never reward animals in ambiguous trials” strategy. Note that results for both the “always” and “never” reward strategies are quite similar to those of the random reward strategy shown in Fig 3C, 3D. (E, F) Results from an RL agent simulation in which reward is based on the ground truth perceptual biases, as in Fig 3E, 3F. The only difference is that, in this simulation, the range of object directions was symmetrical around the true perceptual bias for each of the contextual conditions specified by optic flow. In this case, decision criteria remain the same across contexts, indicating that the small separation observed in Fig 3E results from stimulus range effects. Source data for S3 FigA– S3 FigF are available in “simulation_data/Figure3&FigureS3.mat” at https://doi.org/10.5281/zenodo.15341390. (TIFF) [file pbio.3002764.s003.tif]

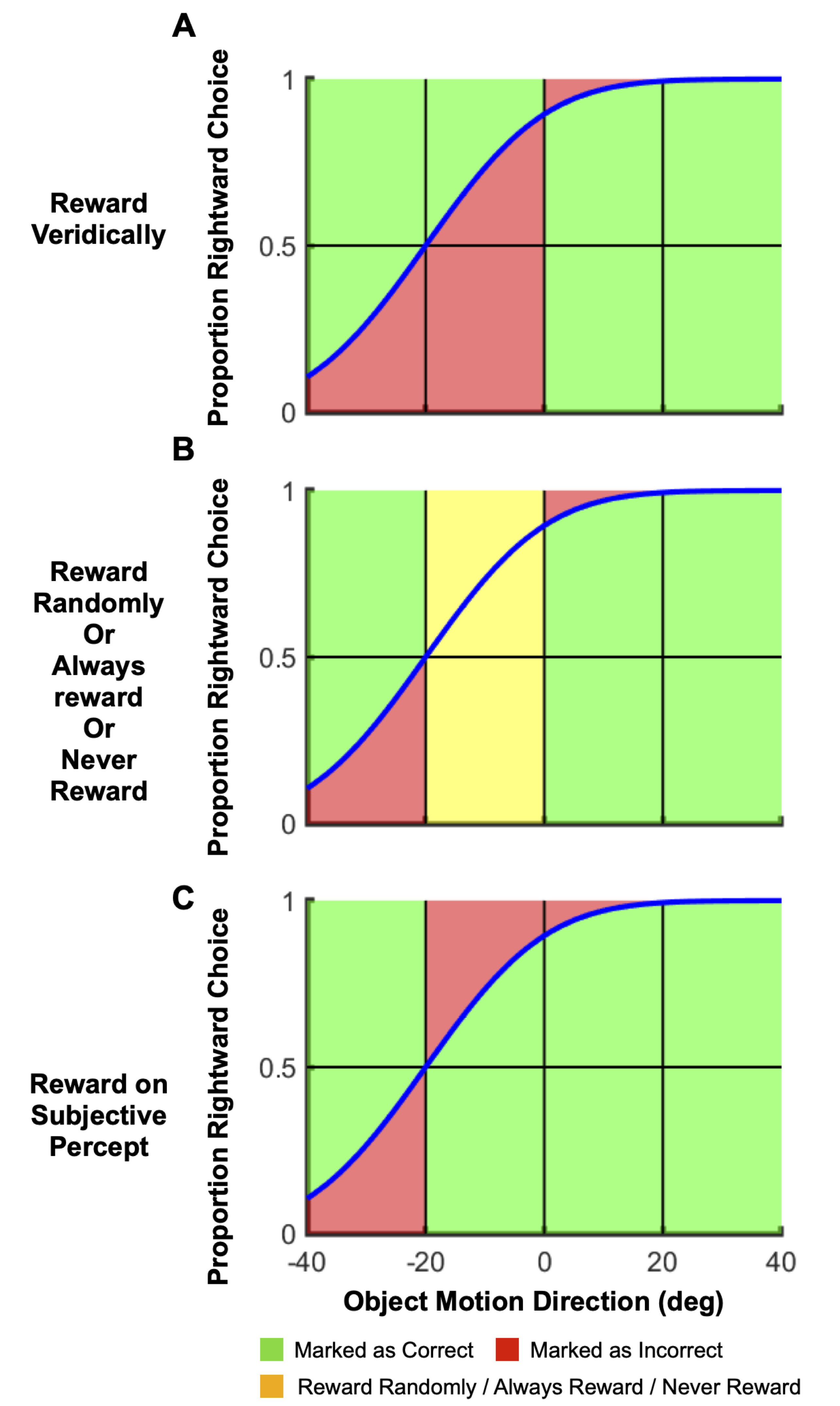

Supplement: S4 Fig — The blue curve represents an example psychometric curve, with perceptual bias P = −20 deg and slope S = 16. Shading indicates the proportions of trials that are scored as correct (green) or incorrect (red), as well as trials that are rewarded differently in different methods (yellow). (A) Rewards are based on veridical stimulus value, such that the reward boundary is at zero object direction despite the perceptual bias. (B) Yellow shading indicates a range of stimulus values for which it is assumed that the “correct” answer cannot be known (i.e., there is an illusion). Within this range, rewards are delivered randomly, always, or never. (C) Rewards are based on the animal’s subjective percept, such that the reward boundary is aligned with the true perceptual bias of P = −20 deg. Note that the animal will receive more total reward (less red area) as compared to the veridical reward strategy. (TIFF) [file pbio.3002764.s004.tif]

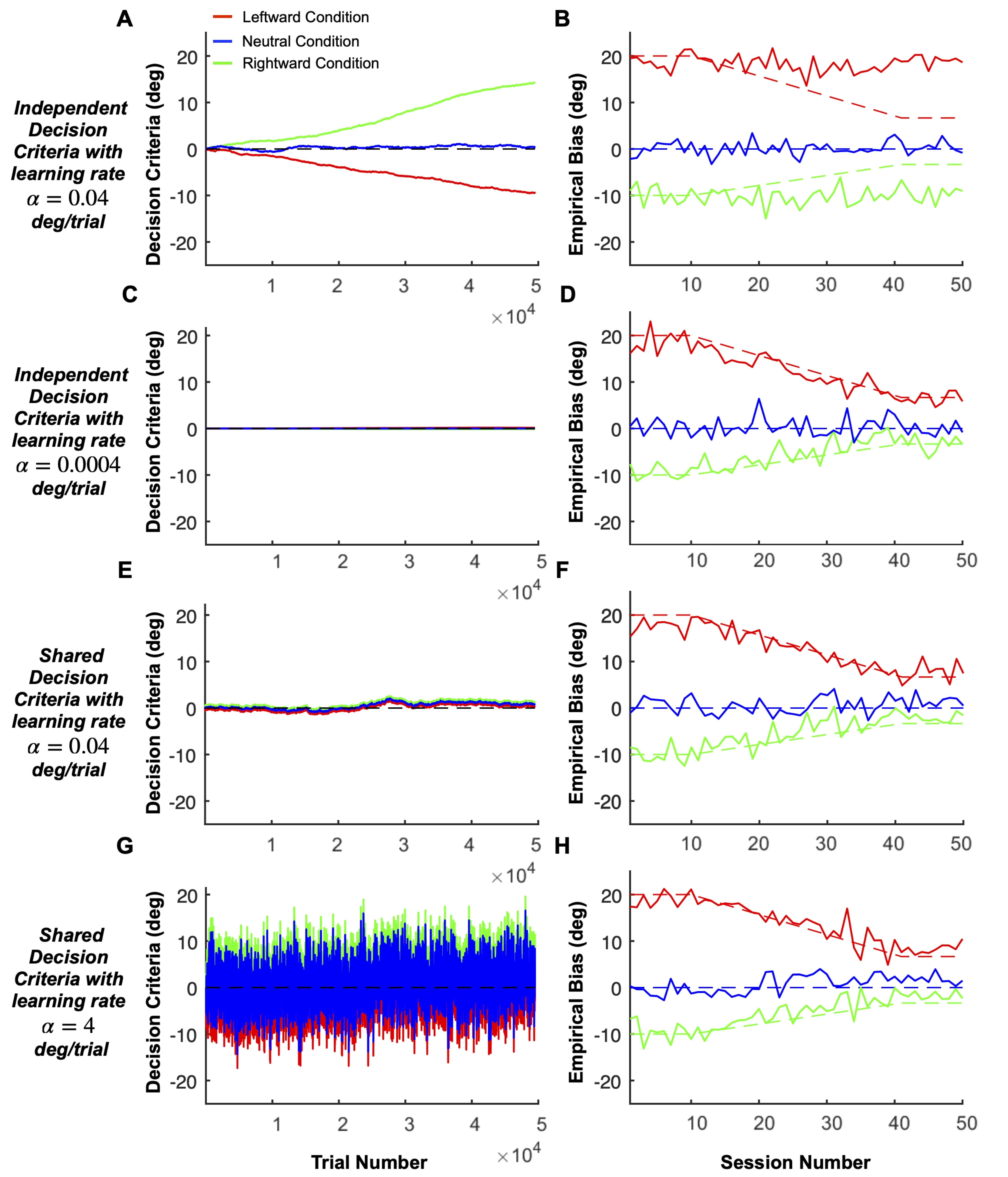

Supplement: S5 Fig — Each panel shows 50 simulated sessions, with each session comprising 990 trials. (A, B) The RL agent adjusts independent decision criteria for each optic flow condition, with a moderately fast learning rate of 0.04 deg/trial. (A) Solid curves depict the learned decision criteria across the three contexts: leftward (red), rightward (green), and neutral (blue) self-motion. The dashed black line (barely visible behind the solid blue line) represents zero decision criterion. (B) Solid curves show the estimated empirical biases in the three self-motion conditions. Red and green dashed lines show how the ground truth perceptual biases change over time, starting at +20 and -10 deg for leftward and rightward self-motion, respectively, and decreasing linearly to 20/3 and −10/3 deg before stabilizing. In this case, the RL agent could adjust its decision criteria faster than our method could track the changing perceptual biases, causing a mismatch between the measured (solid) and true (dashed) biases. (C, D) Same as (A, B), but with the RL agent having a much slower learning rate of 0.0004 deg/trial. In this case, our method accurately tracks the changing perceptual biases. (E, F) Same as (A, B), but with an RL agent restricted to having a single decision criterion that is shared across contextual conditions. Since our method estimates perceptual biases independently of any decision biases that are shared across conditions, it performs effectively in this scenario. (G, H) Same as (E, F), but with a much faster learning rate of 4 deg/trial. If the decision criterion is shared across contexts, our method works well even with very fast learning rates. Source data for S5 FigA–S5 FigH are available in “simulation_data/FigureS5.mat” at https://doi.org/10.5281/zenodo.15341390. (TIFF) [file pbio.3002764.s005.tif]

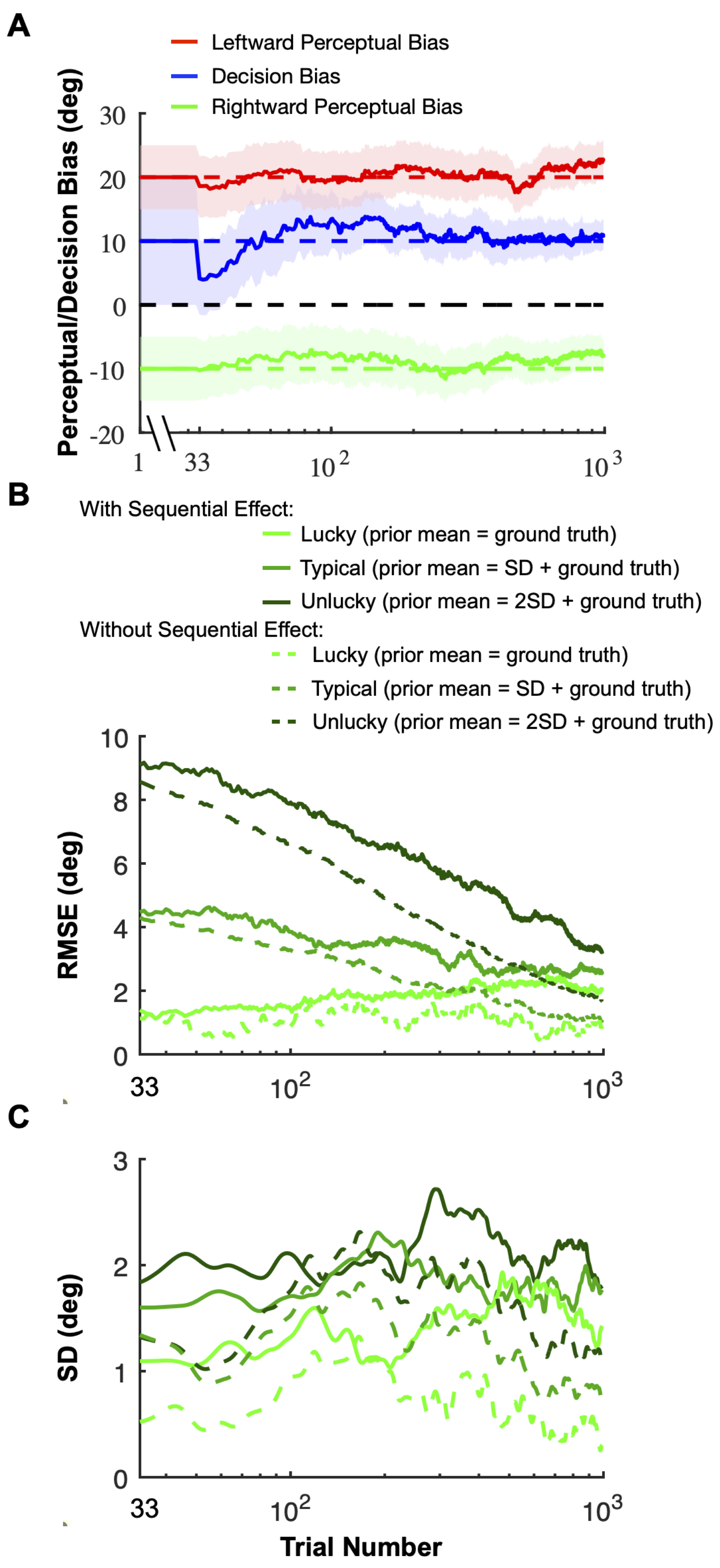

Supplement: S6 Fig — (A) A ground-truth simulation very similar to Fig 6A, 6B, but with built-in sequential choice effects. On each simulated trial, the agent is given a 25 % probability of using a win-stay-lose-shift strategy, repeating the previous choice when rewarded and switching to the alternative choice when not rewarded, regardless of the stimulus values. Note that our method still tracks the ground-truth perceptual biases in the presence of the choice history effect. (B) Solid curves: Average root mean square error (RMSE, y-axis), across 20 simulations, in estimating perceptual bias in the rightward self-motion condition, plotted as a function of trial number. Results are shown for three different prior mean values: 0, 1, and 2 standard deviations (SDs) away from the ground truth perceptual bias (from light to dark green, respectively). Dashed curves: 20 simulations replotted from Fig 5C (matching the repetition number used for the solid lines), which didn’t include any sequential choice effects. When a sequential effect is present, the RMSE shows a slight increase but remains relatively small, indicating that the model’s performance is still adequate. (C) Analogous result to panel B, but showing the average standard deviation (SD) of perceptual bias estimates as a function of trial number. Again, performance is similar, but SDs are somewhat larger in the presence of choice history effects. Source data for S6 FigA–S6 FigC are available in “simulation_data/FigureS6A.mat” and “simulation_data/FigureS6BC.mat” at https://doi.org/10.5281/zenodo.15341390. (TIFF) [file pbio.3002764.s006.tif]
