# Supplementary material for: Rewarding animals based on their subjective percepts is enabled by online Bayesian estimation of perceptual biases
Source: PLoS Biol. 2025 May 20;23(5):e3002764. doi: 10.1371/journal.pbio.3002764 (PMC12129325; doi:10.1371/journal.pbio.3002764)
Supplement: Attachment — Submitted filename: DoLe_HaDe_Response2Reviews_rewardPercept_PLOS_Biology.pdf [file pbio.3002764.s007.pdf]

Reviewers' comments and suggestions are presented in black. Our responses to the reviewers are highlighted in blue. The corresponding line numbers in the revised manuscript with change tracking where the reviewer's comments were addressed are indicated in red.

Reviewer #1: In their manuscript "How to reward animals based on their subjective percepts: A Baysian approach to online estimation of perceptual biases", Dong, Lengyel and colleagues have developed an elegant method to update the reward for each choice in a perceptual decision task based on the estimated perceptual bias of the animal. The manuscript is relevant to the important question about the biological nature of perceptual biases and how we can study these in animals. The proposed reward method is grounded in Bayesian methods, highly principled and constitutes an important improvement on current methodology. However, it relies on number of prior assumptions about the nature and dynamics of perceptual and decision biases and how they can be measured. The method will be very useful to the community, but I think this manuscript is a missed opportunity to explore more fundamentally the neurobiological nature of the different biases in perceptual processing.

Thank you for the positive assessment and the helpful comments below. We have now added several new simulations and additional analyses (discussed in detail below) that shed important additional light on the dynamics of decision and perceptual biases, as well as the clarifying assumptions.

1.1. Perceptual biases are important because they shift the most sensitive parts of the psychometric functions in different contexts, allowing the animal to maintain discrimination performance. If you so will, they allow to probe with heightened sensitivity along a spectrum. The goal of the introduced reward method is to counteract a diminishing perceptual bias when the animal is not rewarded for reporting it over many trials and sessions. A phenomenon the authors have reported before. With their Bayesian approach to rewarding the animal's choices, the authors try to stabilise the accurate report of a perceptual bias. What is not addressed is whether their method stabilises the report of a persisting perceptual bias or whether their reward schedule is necessary to maintain the perceptual bias itself. This question is important for our understanding of the neurobiological basis of perceptual biases and the effects of reward on perception. It also has implications for the reward method introduced here and how it can and should be applied.

In general, a key goal for animal psychophysics is to design a reward schedule such that reward maximization aligns with reporting the desired perceptual quantity. How to do so in a situation when the animal's subjective percept is different from a physical, experimenter-controlled, stimulus variable (the "perceptual bias" being the difference between the two) is the challenge that our method addresses. When one observes diminishing response biases over time in empirical data (e.g., Peltier et al. 2020), one can generally not know whether the animal's perceptual biases have changed or whether they have altered their decision criteria, in a context-specific manner, to compensate for the perceptual bias and receive greater reward. Our reward method stabilizes the *reports* of existing perceptual biases without altering or inducing new perceptual biases. To validate this, we ran additional simulations using a reinforcement learning (RL) agent that has complete freedom to adjust its decision criteria across the three contextual conditions based

on feedback (see Lines 153-208 in the Results, and 880-935 in the Methods). As shown in Fig. 3G&H (new figure introduced on Line 199), when the RL agent had pre-existing perceptual biases in the two contextual conditions, our reward strategy preserved the correct reporting of these biases across the simulated sessions. In contrast, other reward strategies led to substantial changes in decision criteria and resulted in response biases in the simulated data that deviated substantially from the true perceptual biases of the RL agent. (Note that, in the manuscript, we refer to response biases observed in psychometric curves derived from empirical or simulated data as "empirical biases".)

1.2. If the Bayesian reward method simply stabilises the report for a perceptual bias that persists, then the animal can shift their behavioural response criterion independently for different trials in different directions in the leftward and rightward bias inducing contexts. This contradicts at least some reports of what animals can do when different reward criteria are explicitly signalled by a change in the stimulus (e.g. Cicmil, Cumming, Parker, Krug 2015, elife, visual perturbation experiment).

With our approach, animals receive rewards for reporting their naturally occurring perception – whether biased or not –- removing any incentive to alter their decision criteria to increase rewards. Our new simulations (Fig. 3G&H, see on Line 199) demonstrate that this approach works, such that the RL agent's decision criteria remain stable and near zero across conditions when using our reward method.

Our RL agent is given the power to independently adjust its decision criteria across contexts, and thus presents the greatest challenge to different reward schemes (see Lines 153-208 in the Results, and 880-935 in the Methods). The extent to which animals could actually shift their decision criteria independently across contexts is likely to depend on the task and the stimuli. Importantly, in either case, our method works. If animals can independently adjust their decision criteria across contexts, our method eliminates their incentive to do so by estimating their perceptual bias and aligning the reward boundary to that estimate. Conversely, if animals cannot independently adjust their decision criteria, our method still removes any incentive to alter decision criteria altogether (see Lines 754-767 in the Discussion).

1.3. However, if this is indeed the case, how reliable is then report from the prior or current sessions to infer perceptual and decision biases and how robust is the method in supporting the "real" percept when the experimenter gets it qualitatively not just quantitatively wrong (e.g. no bias if there is one)?

If the experimenter's initial estimates of an animal's perceptual biases are inaccurate, our algorithm will converge quickly (typically within one session) to the true perceptual biases, as shown in our ground truth simulations. In Fig. 5C (previously Fig. 4), the dark green line represents a case for which the prior is set 2 standard deviations from the true bias. Even in this extreme scenario, the algorithm accurately converges within ~1,000 trials. Importantly, a non-existent perceptual bias in our model is simply represented as zero, making it not qualitatively different from any non-zero bias (see Lines 611-626 in the Discussion).

1.4. In contrast, if the reward schedule is necessary to maintain the perceptual bias itself, then the new reward method potentially actively alters visual processing of the stimulus. In

this context, the literature showing effects of expected reward on neuronal signalling in visual perceptual decision tasks is relevant (e.g. Baruni, Lau, Salzman 2015 Nature Neuroscience; Cicimil, Cumming, Parker, Krug 2015 elife) and should be discussed.

As noted earlier, our method does not artificially induce or maintain a perceptual bias that would otherwise not be there due to the contextual modulations. If no perceptual bias exists, our method quickly estimates a zero perceptual bias and rewards accordingly, without inducing a perceptual bias. Our simulations demonstrate that our method correctly infers the intrinsic perceptual bias (Fig. 3G&H, see on Line 199), and can even track it if those changes happen slowly (Fig. S5C&D on lines 1168).

We appreciate the reviewer's suggestion to include Cicmil et al. (2015). This reference has been incorporated into the manuscript when we discuss whether animals can independently adjust their decision criteria across contexts (see Lines 754-768 in the Discussion).

1.5. An important experimental test would be whether - using this new Bayesian reward method - the report of a perceptual bias in an animal in which the report of a perceptual bias has disappeared over time can be re-established. If the method does what it is proposed to do according to the authors, then this reward schedule would recover a persistent but unreported perceptual bias. But if the perceptual bias itself has disappeared, then the dynamic updating of the reward schedule should not induce a new perceptual bias.

Our reward method only stabilizes the reporting of existing perceptual biases; it does not induce a bias that was not already present, nor does it eliminate existing perceptual biases. Therefore, if empirical biases have reduced over time due to adjustments in decision criteria (as in Fig. 3A-D, Line 199), our method cannot recover those lost biases. However, it may be possible to use our method in combination with additional control conditions to estimate the animal's decision criteria under contextual modulation. By accounting for the decision criteria, one might be able to estimate the true perceptual biases (see Lines 769-777 in the Discussion).

1.6. There a number of assumptions here in order for the reward schedule to work.
All biases are stationary within a session. There is plenty of evidence that this is not the case; the authors cite some themselves. Can the authors estimate explicitly up to what point their model is robust, in other words what temporal variability in the bias they can tolerate?

If biases fluctuate within a session, our method will estimate the average perceptual biases in the session. This is no different from most psychophysical methods. If you compute a psychometric function using all trials in a session, then your estimates of sensitivity and bias are effectively the average of the session (see Lines 709-710 in the Discussion).

Moreover, our assumption of stable perceptual biases within a session aligns with relevant literature. In perceptual learning studies substantial changes in performance typically require extensive training over 5-10 days (Bekrater-Bodmann et al., 2012; Cretenoud et al., 2021; Dosher & Lu, 2017; Fahle & Poggio, 2002; Jeter et al., 2009; Karni et al., 1994; Karni & Sagi, 1993; Miyamoto et al., 2016; Sagi, 1994; Watanabe & Sasaki, 2015) while decision biases may vary from session to session and can gradually shift within a single session (Ashwood et al., 2022; Fan et al., 2018; Feng et al., 2009; Gao et al., 2011; Rorie et al., 2010) (see

Lines 700-720 in the Discussion). As seen in Fig. 5C, our ground-truth simulations show that one session is sufficient to refine even a highly inaccurate initial estimate of perceptual bias, indicating that our method can accommodate perceptual biases that shift gradually over several sessions. Moreover, our new simulation shows that our method can track a gradually changing perceptual bias across sessions (Fig. S5C&D, Line 1168). Additionally, while our model assumes a stationary decision bias within each session, it remains robust to gradual changes within a session (see Fig. 5B).

Finally, to clarify the necessary assumptions: any method aiming to separately estimate perceptual and decision biases in 2AFC tasks must fundamentally assume that these biases are distinct. However, how the biases are defined can vary across experimental paradigms. The method that we propose here is adaptable and can be applied alongside other task and decision-making models, including those that account for nonstationarities or sequential effects. For studies examining other types of biases or paradigms where rapid shifts in decision biases may occur—factors that could complicate perceptual bias estimation—our model could be extended to incorporate nonstationarities in decision biases (see Lines 687-699 in the Discussion).

1.7. One bias that is ignored are sequential effects in decision-making, e.g. Lueckman, Macke, Nienborg 2018 J Neuroscience. Reward history has been implicated as a factor in this. The authors should discuss potential interactions with the effects of their reward schedule.

Thank you for this suggestion! We have now tested this and numerically found that they only moderately increased variability & uncertainty for reasonable amounts of serial dependencies in our method's estimates (Fig. S6C, on Line 1189). This is not surprising since serial dependencies do not have a *systematically* different effect on the different contextual conditions when these are interleaved. However, the presence of serial effects increases the uncertainty in the estimates, as serial dependencies violate the model's assumption of trial independence (Fig. S6C, on Line 1189). Nevertheless, this increased uncertainty is moderate and does not significantly impact the accuracy of our method for realistic amounts of serial correlations (see Lines 741-753 in the Discussion, and 1006-1011 in the Methods).

1.8. There need to be two contexts that induce perceptual biases in equal and opposite directions. How can the method be adapted of the perceptual bias is just in one direction or might be unequal?

We apologize for not making this clearer in the manuscript. In our method, the perceptual biases across the two contextual conditions are NOT required to be equal and opposite (we mentioned this case only as an example of prior work: Linares et al. 2019). The model includes three contextual conditions: two with perceptual biases in opposite directions but differing in magnitude, and one neutral condition with no expected perceptual bias (only decision bias). Figures 5 and 6 illustrate these differing perceptual biases for the 'rightward' and 'leftward' conditions, as was the case in the previous version of the manuscript (see Lines 245-275 in the Results).

The minimal requirement for our method to work is a single condition involving contextual modulation. This requires interleaving a neutral control condition in which no contextual modulation is present (and thus no perceptual bias is expected). In this case, the empirical bias measured in the neutral condition provides an estimate of the animal's decision bias. Subtracting this decision bias from the empirical bias measured in the contextually modulated condition yields the perceptual bias. Thus, while the main examples that we present have 3 conditions (one neutral and two contextual conditions), our approach only requires one neutral condition and one with contextual modulation (see Lines 250-254 in the Results).

1.9 There are a number of points where the methodological choices for determining reward trial-by-trial could be more explicitly discussed for the non-expert reader.
The manuscript (Figure 2) states that effectively the reward boundary is shifted (trial-by-trial) and then the animal's choice rewarded depends on which side of the boundary the response falls on for a given stimulus and cortex. Why is there not an element of probabilistic reward near the criterion, because near-threshold choices would not yield choices all in one direction? Would it potentially improve the outcome if the reward probability was explicitly altered?

In theory, stochastic rewarding reduces the learning signal and slows down the learning process, even when the stimulus is near threshold (e.g., Campbell et al. IEEE 2015; Romoff et al. CoRL 2018; Corazza et al., AAAI 2022; Cai et al. arXiv 2023). For this reason, we (similar to other studies using 2AFC tasks for fine discrimination) chose to avoid introducing any stochasticity in the rewards. We extended the Results (Lines 316-320) and Methods (Lines 847-879) sections to clarify our trial-by-trial reward algorithm explicitly.

1.10. It would be helpful to see in the methods a full equation that calculates the reward boundary and how it is updated trial-by-trial, rather than just the individual parts of the equations spread out.

Thank you for highlighting this point. We have now also included a more detailed and step-by-step explanation of how we used our method to administer rewards for our monkey in the Methods section on Lines 847-879.

1.11. There seems to be no element of discounting choices on trials earlier in the session through differential weigthing as the session progresses. Is this an active choice and why?

Our aim was to address the common scenario of studying average perceptual biases in animals under specific contextual modulations. The average perceptual bias can optimally be estimated by weighting all trials equally. However, our algorithm proved robust to slow non-stationarities, suggesting an implicit underweighting of the prior, effectively discounting earlier trials (see Fig.5B and FigS5C&D). Additionally, our method can be easily extended to incorporate explicit discounting of past trials if desired (see Lines 700-740 in the Discussion).

1.12. Was the amount of reward per trial flat throughout the experiment and the simulations? How would the outcome be affected by a reward escalator typically used when working with monkeys, i.e. where the amount of reward is determined by the number of preceding correct trials?

During training sessions with monkeys, rewards were progressively increased to sustain motivation on an ad-hoc basis. The absolute amount of reward does not affect our method. Our method is flexible and can also accommodate alternative reward schemes, such as a reward escalator, where the reward amount increases based on the number of consecutive correct trials (see Lines 847-879 in the Methods). If there is a concern about a problematic interaction between a bespoke reward scheme and our method in a specific application, our RL simulation code can be used to easily check for such effects.

Minor points:

- figure 5A: the panel and elements are to small to see adequately on a printed out version. Online magnfication reveals poor resolution of, for example, the white elements in the receptive field.

We apologize for this and have now made panel A larger.

- figure legend for Figure 5C refers to a "fat" prior. Should be flat.

Thank you for pointing out this mistake. We corrected it.

- the "flat" prior is not defined in the manuscript

Thank you! We define it now on Line 343 when we first use this term:
"..uniform prior, equivalent to maximum likelihood estimation.."

- detailed methods on the visual stimulus and task (luminance, size of stimulus, eye tracking etc.) would be useful (or a reference to another paper in the methods specifying these parameters).

We extended the description of the experiment in the Methods adding these details on Lines 1013-1024.

—--

Reviewer #2: Dong and colleagues proposed a new method of adaptively rewarding monkeys for experiments involving perceptual biases, based on online estimates of the perceptual bias. They used a Bayesian method to obtain an estimate of perceptual bias for each trial, incorporating and updating a prior that was initially derived from previous sessions, and set the reward boundary that combined the actual stimulus info and the estimated perceptual bias. They used simulations to demonstrate the robustness of their online estimation method and showed that, in one monkey, training with this new reward method did not induce a gradual reduction in perceptual bias.
This study adedresses an important issue in studies of animal cognition, where it is often difficult to interpret changes in a behavioral report in relation to changes in perception alone. The manuscript is well-written and the analyses were performed rigorously. The results are convincing for estimating perceptual bias. However, I am not fully convinced that the online-estimate-based reward method is necessary in practice.

Thank you for your positive feedback on the technical soundness of our work and your helpful comments. We hope to convince the reviewer that the reward method we propose is necessary and should be considered best practice for studying perceptual biases across a wide range of applications.

Major comments
2.1. This study was motivated by a concern that existing rewarding methods might cause apparent decreases in perceptual bias because an animal could in theory learn higher-order strategies to compensate for the biasing conditions.

The issue of how to reward animals on illusion (or expected perceptual bias) trials is critical in all animal studies investigating biased perception. Many studies briefly address the challenge of developing effective reward strategies in these trials to prevent animals from adopting compensatory response strategies when they aim to maximize their rewards (e.g., see reviews by Beran & Parrish, 2022; Santaca et al., 2021; and empirical studies such as Agrillo et al., 2015; Agrillo et al., 2014; Clara et al., 2006; Barbet & Fagot, 2002, 2007; Huang et al., 2002; Klingenhoefer & Krekelberg, 2017; Schielke & Krekelberg, 2021; Krekelberg et al., 2003). Many of these studies acknowledge that their chosen solutions (e.g., always, never, or random rewards) are suboptimal, as they can affect animals' responses, thereby limiting the number of illusion or expected perceptual bias trials they can use in the experiment.

Although only a few studies have provided empirical evidence of this issue (i.e., diminishing perceptual biases over time), we cite more studies in the revised manuscript that explicitly demonstrate the problem in the domain of motion perception (Peltier et al., 2020), face distortion illusion (Gao et al., 2024), and flash lag illusion (Subramaniyan et al., 2013) on Lines 42-48 and 59-60 in the Introduction.

Furthermore, we now include in the manuscript a range of numerical simulations of an RL agent (modeling the animal) that explicitly show the detrimental effect of these various suboptimal strategies used in prior work on the accurate estimation of perceptual biases (see our response to the Reviewer's point 2.3 below).

Our work addresses this challenge and, to our knowledge, is the first to propose an optimal solution.

2.2. As an example, the authors cited one set of results from their previous experiment (Fig 11 of Peltier et al 2020), stating that the perceptual bias decreased over time. However, I am not sure that this is an accurate description of the figure. For example, during training, monkey M increased its flow-parsing gain and maintained that gain through >30 sessions (estimated by eye; experiments 1 and 2). The gain was only decreased, quite abruptly, when a new experimental condition was introduced and then was maintained for the rest of the study. Monkey P decreased the gain during training, but then maintained it over a year without further reduction after the initial decrease. These results do not unequivocally support the authors' concern about a gradual decrease in perceptual bias with the existing reward methods.

In regard to the Peltier et al. (2020) study, the reviewer is correct in noticing that there were more rapid changes in flow parsing gain initially. However, these were followed by continued gradual reductions. For monkey M, during the 20 neural recording sessions that followed training (blue symbols in Figure 11A of Peltier et al. 2020), there was a significant decrease in flow parsing gains (linear regression: Pearson's $R = -0.66$, $p = 0.0015$).

That said, we agree with the reviewer that these empirical data are not the best way to justify the need for our approach. Thus, as suggested by the reviewer and described below, we implemented a reinforcement learning agent, and we show clearly that previously used reward methods lead to large errors in inferring perceptual biases, whereas our method does not. We think this provides a much stronger demonstration of the need for our approach, and thus we no longer rely much on interpretation of the Peltier et al data (see Lines 543-545 in the Results and Lines 565-568 in the Discussion).

2.3. The authors' ground-truth simulations showed convincingly that they can estimate perceptual bias online and their estimation method outperforms Psignifit. However, given that the goal of the paper is to devise a reward method to counter compensatory adjustments in decisions, it is surprising that the authors did not try to recapitulate the learning process in their ground-truth simulations to demonstrate how their method compares with alternative methods (e.g.., random rewards, a fixed boundary at the objective 0, a fixed boundary based on the informed prior, online estimates based on some wrong values of prior mean/sd, and online estimates based on Psignifit, etc.). It would also be helpful to understand better how much errors in perceptual bias estimates propagate over trials/sessions through suboptimal reward boundaries.

This is an excellent suggestion, which we have now implemented for the revision of the manuscript (see Lines 153-208 in the Results, and 880-935 in the Methods). We performed simulations using a reinforcement learning (RL) agent that can adjust its decision criteria in three contextual conditions based on feedback (following Law and Gold, 2009; Williams, 1992). We then compared the reward strategy used in previous studies with the optimal strategy (i.e., rewarding based on the **ground truth** perceptual biases) and with our approximation of the optimal strategy (i.e., rewarding based on the **estimated** perceptual biases). Our findings clearly show that previous reward strategies lead to a large underestimation of perceptual biases in the contextual modulation conditions across

sessions (see Fig. 3A-D, Line 199 and Fig. S3A-D, Line 1168). This reduction occurs because the RL agent adjusts its decision criteria in response to feedback, thus compensating for its inherent perceptual biases to maximize rewards. In contrast, when rewards are allocated based on the true perceptual biases in conditions with contextual modulation (i.e., the reward boundary matches the inherent perceptual biases), the RL agent barely adjusts its decision criteria (see Fig. 3E&F, Line 199), suggesting that rewards can be maximized without any change in the decision criteria. Consequently, the estimated empirical biases remain stable at their initial values. Thus, crucially, these new simulations demonstrate clearly that one cannot reliably estimate perceptual biases using conventional reward methods if the learner has some capacity to adjust their decision criteria.

2.4. For simulations, it was assumed that the decision bias D was constant from trial to trial with an interleaved-context design. However, this raises the question of why/whether monkeys would still be able to compensate for perceptual biases with such a task design. How much of the stable behavior is due to the interleaved-trial design versus the reward method?

If by "stable behavior" the Reviewer meant preserving a stable measurable perceptual bias, this appears to depend entirely on the reward method. First, in the study by Peltier et al. (2020), an interleaved-trial design was used; however, this design did not prevent the animals from compensating for their inherent perceptual biases, and thus the measurable bias continued to decrease despite the interleaved design. Second, in our new simulations, we implemented a 2AFC task with randomly interleaved conditions, and whether the perceptual bias diminished over sessions or not depended solely on the reward method (Fig. 3, Line 199).

Regarding decision biases or criteria that differ across contexts, our method eliminates any incentive to develop distinct decision biases for the three conditions to maximize rewards. Decision biases unrelated to reward maximization are unlikely to vary across interleaved contextual conditions. For decision biases or criteria that are shared across contexts, our method is well-suited to handle them within the interleaved design (see Lines 687-720 in the Discussion).

2.5 The results in Figure 5 need clarifications. First, for the lower example, there seems to be a substantial discrepancy between the decision bias (near zero, estimated by looking at the blue psychometric curve) and those in C. How much does such a discrepancy matter?

There is no discrepancy between the psychometric curves in Fig. 6B (previously Fig. 5B) and the curves in Fig. 6C (see Lines 433-443 in the Results for a revised explanation). The psychometric curves in Fig. 6B display the average empirical response biases using data from the entire example session, while the curves in Fig. 6C show the 'running' estimates of the average perceptual and decision biases using only data up to each trial shown on the x-axis. These two figures can be aligned when comparing the average empirical response biases derived from the final estimates of the perceptual and decision biases (y-values at the last trial on the x-axis in Fig. 6C) to the fitted empirical response biases in Fig. 6B. This alignment is especially clear with the decision bias, as it directly translates to the empirical response bias on the psychometric curve in the neutral condition. The final point in Fig. 6C,

showing the decision bias in the neutral condition, does match with the empirical bias in the psychometric curve's neutral condition in Fig. 6B.

2.6. Second, it is not clear why flat prior has higher variability than informative prior only for the rightward condition.

What the reviewer picked up on is not a consistent aspect of the data. The variability in all bias estimates is consistently greater when using a flat prior compared to an informative prior. This characteristic, a universal aspect of Bayesian inference, is also evident in our simulations in Fig. 7A: the wider the prior, the greater the uncertainty around the bias estimates. In the example session at the top of Fig. 6C, the animal happened to have a strong initial bias in the Rightward condition over the first ~100 trials, which we think was a quirk of this particular session (see Line 459 in the Results).

2.7. Third, how much of the dynamics in perceptual and decision bias were due to estimation versus monkey changing its behavior based on the reward method?

Since we do not know the 'ground truth' biases in real applications, we cannot answer this question with any certainty. However, our new simulations show that our reward scheme does not induce new perceptual biases; it maintains inherent biases that predate the experiment. Fig. 3G&H demonstrates that our method maintains existing perceptual biases. Additionally, animals' decision biases may adapt more quickly than perceptual biases in response to feedback, which is why we use the interleaved neutral condition to track decision biases shared across context independently of perceptual biases. Please also see our response to the first two points from Reviewer 1 (1.1 & 1.2.).

2.8. Figure 6 is supposed to support the idea of pooling multiple sessions to get better prior estimates. However, while panel A shows that a larger prior width is beneficial (the mean bias estimate is closer to ground truth at larger prior width), panel B shows the SD decreases with increasing number of sessions included. This apparent contradiction is likely due to better estimates of *mean* prior with more sessions, which is unfortunately not shown.

Panel A in Fig. 7 (formerly Fig. 6) illustrates the classic bias-variance trade-off: narrower (overconfident) mismatched priors result in low-variability estimates that are biased away from the true perceptual bias values, while wider (underconfident) mismatched priors lead to less biased but more variable estimates. In this simulation, wider priors are beneficial only because they are centered around incorrect bias values.

Panel B shows that uncertainty in the bias estimates decreases across sessions, which is only advantageous if the estimates also converge toward the "ground truth." However, in this panel, our method is applied to real data where the ground truth bias is unknown, so we could not include the suggested figure showing estimates converging to the true bias values.

2.9. I appreciate the practical difficulty of testing different reward methods on monkeys. However, it remains that the data from 50 sessions in one monkey are not strong evidence that the critical determinant is the new reward method (especially given that monkeys may also maintain stable biases with existing methods, e.g., monkey M in Peltier et al. 2020).

We agree with the reviewer that data from a single monkey does not provide strong evidence on its own. However, our new simulations with known ground truth do. Our new simulations, which compare different reward strategies, clearly demonstrate that all previous methods result in diminishing measurable biases (Fig. 3, Line 199 and Fig.S3, Line 1144) if the animal can adjust its decision criteria to maximize rewards. With these new simulations, we no longer rely on the empirical data to demonstrate the approach works; however, we still include these data as they provide some additional supporting evidence. Our proposed method offers an optimal solution to a problem highlighted in numerous prior studies (see, e.g., reviews by Beran & Parrish, 2022; Santaca et al., 2021, and empirical studies by Agrillo et al., 2015; Agrillo et al., 2014; Clara et al., 2006; Barbet & Fagot, 2002, 2007; Huang et al., 2002; Klingenhoefer & Krekelberg, 2017; Schielke & Krekelberg, 2021; Krekelberg et al., 2003; and demonstrations of decreased perceptual bias in Gao et al., 2024; Peltier et al., 2020; Subramaniyan et al., 2013).

Minor:

- At the beginning of a session, the authors gathered data from the first 33 trials. How were these trials rewarded?

In these trials animals were rewarded based on the result from previous sessions. We added this information in the Methods section of the manuscript (Lines 847-879).

- Line 285, the white dashed line in Figure 5A was not visible.

Thank you for noticing this. We made subplot A larger and we increased the resolution for all figures in the paper.

- Pg 12, Figure 5 legend. line 5 from bottom. "fat" -> "flat"?

Thank you for pointing out this mistake. We corrected it.

Reviewer #3: This paper describes a novel psychophysical modeling framework for separating out perceptual and decision biases in psychophysical experiments, along with a closed-loop method for rewarding animals based on their percepts (while continuing to penalize them for decision-related bias). This overcomes an important limitation of previous work, which (if it relies on rewarding animals purely on the basis of "ground truth" stimuli) will effectively train an animal to over-ride those perceptual biases that scientists would like to analyze. The paper provides an extremely elegant solution to a compelling problem. I enjoyed it immensely and feel it will make an outstanding contribution to the literature. I have only two general comments and a number of more detailed comments for improving readability and clarity.

Thank you for the positive assessment of the manuscript and the helpful comments.

General comments:

3.1. Given that the method needs to run quickly enough to provide an updated estimate of the perceptual bias during the inter-trial interval, I think it's important to show some kind of speed comparison. How long does it take to run (and how does it scale with dataset size?) (Apologies if this was mentioned somewhere in the paper, but I did not see it!)

Our method runs in about 1-3 seconds. This means that after a trial ends our algorithm needs about 1-3 seconds to compute the optimal reward. The computation time increases with the data size and the number of posterior samples, but even with typical animal training paradigms involving 500–5000 trials and 1000–10,000 samples, delays remain within the 1–3 second range.

Also note that (a) this time will be reduced on faster computer hardware (we currently run this on a Dell T3500 workstation from 2010), and (b) the time required can be ameliorated by reducing the number of MCMC-samples used to perform the (approximate) computations. Reducing the number of samples is equivalent to adding a little bit of variability to the reward schedule - as suggested by reviewer 2.

We included this information in the Result on Lines 311-315 and in Methods section on Lines 828-848.

3.2. A second comment is that I feel the authors have missed an opportunity to show the superiority of their method in a setting where the animal updates its decision strategy based on the rewards it receives. The paper's motivation (eg made explicit in Fig 2B) leans heavily on the idea that rewarding an animal based on the "veridical" or "world" stimulus will lead it to learn to override its perceptual biases and simply make reports that maximize its probability of reward. (In other words, it will learn to give unbiased responses). However, the simulations did not consider any scenario where this occurs; in the simulated datasets, the animal had fixed values of the perceptual biases P_R and P_L and either fixed or sinusoidally modulated values of the decision bias D.

It seems to me that it would be a stronger validation to consider a setting where the animal updates its decision parameter according to some kind of reinforcement learning rule to

maximize the reward. (For example, D could be updated according to a policy gradient learning rule... or perhaps some kind of Q learning?). One could then explicitly compare the efficacy of the different reward strategies proposed: rewards based on true stimulus, random rewards or no rewards for stimuli that elicit biased percepts, or rewards based on the authors' proposed method. This would also allow them to show that their method leaves the animal's original perceptual bias more intact than alternative methods!

(Note I recommend adding such a simulation, but I would not consider it essential).

This is an excellent suggestion, and we have performed the suggested simulations (see Lines 153-208 in the Results, and 880-935 in the Methods). Please read our responses to point 2.3. of the second reviewer for details. Indeed, as the reviewer predicted, these simulations of an RL agent demonstrate that our method greatly outperforms other previous reward schemes that have been used (see Fig. 3 on Line 199). We feel that these results greatly strengthen the paper, and appreciate the recommendation.

Detailed comments:

3.3. Sec 3.1 : I'm afraid it isn't clear enough what the task is. (What is the "vertical task reference"? Is this a separate task? It isn't clear what this is or how it relates to the standard task.). All the text says about the task is that it is "object motion perception"; please provide a bit more detail on what the stimulus is and what the animal is expected to do. It also isn't clear to me why retinal velocity and world velocity are different. (If the observer isn't actually moving -- i.e., there are just some distractor dots in the background -- then isn't the retinal velocity the same as the world velocity). The text here feels as though it is written for a reader who is already intimately familiar with the experimental details from some earlier study.

Thank you for your comment. The task in the example in Section 3.1 involves motion direction discrimination around a vertical reference while the observer experiences self-motion simulated by optic flow. In this case, the retinal motion of the target is what is actually presented on the screen, while the "world" motion refers to the motion of the target in the world that would give rise to the retinal motion in the presence of self-motion as implied by the optic flow. This hypothesized "world" motion can be computed by subtracting the optic flow motion from the retinal motion. Note that this example task is just used for illustration (see Lines 97-103 in the Results), and that the task performed by the monkey is a variant that is now described in greater detail in the Methods section (see Lines 1013-1024 in the Methods).

3.4. line 168: "The two perceptual biases can then be computed by subtracting the empirical bias measured in the neutral condition from the empirical biases measured in the other two conditions (Fig. 3B)."

Does this mean you DON'T assume that the two perceptual biases have equal magnitude but opposite sign (i.e., the critical assumption made by the first method?). It would be nice to spell this out, if so. (Also, it would be nice if you follow up later to state whether this assumption holds or not in real data!)

Edit: after reading the following section (which describes the details of the method), I wouldn't describe your method in quite this way. If I understand correctly, it's not that you're using just the neutral condition trials to estimate the get an estimate of the decision-bias and then subtracting it off; you're using data from all trials to infer all the model parameters, right? (This is actually a good thing from a Bayesian perspective-- better than separately trying to estimate decision bias from only 1/3 of the trials). So this is maybe the essence of what's happening, but it might be worth clarifying that this isn't literally the approach you're taking.

Edit #2: after reading 3.3 I see that indeed you don't assume the two biases are symmetric. So it might be worth pointing that out above when you introduce the method!

Thank you for the suggestion. We have clarified these issues in Lines 245-275, where we describe the method involving three interleaved conditions. Please read our response to point 1.8. of the first reviewer.

3.5. 185: First, as commonly done (Prins, 2023;
186 Schutt et al., 2016), we assume that the percentage of making one of the two choices (e.g., a
187 rightward response) follows a binomial distribution with parameter theta denoting the probability
188 of choosing the first response alternative, and parameter n representing the number of trials.

I might suggest describing this as Bernoulli on each trial (instead of binomial over n trials). Here n isn't really a 'parameter' of the model in the sense of parameters you're fitting.

By the same token, I wouldn't refer to theta as a parameter either. (Yes, it's the parameter of the Bernoulli distribution on each trial, but it's not a parameter you're fitting to the data, but rather a quantity derived from the stimulus and model parameters on each trial... you might call it a latent variable). So I would advise being a bit more careful with terminology and definitions. (And as I note below, adding a few equations would also help!)

Thank you for these suggestions. We changed the text accordingly (see Lines 279-281 in the Results, and 793-798 in the Methods).

3.6. 189: we used a
190 cumulative Gaussian distribution as the functional form of the psychometric curve, reflecting
191 the relationship between ✓ and the stimulus value, !, (e.g., object motion direction).

Personally I would recommend putting the actual equation in here (which I think is easier / clearer than trying to say this in words), e.g.,

P(right choice | omega) = theta = Phi ((omega - B) / S),

where B = P + D, and Phi is the cumulative normal distribution function. (Or whatever you have in mind).

There's no need to reserve all equations for the Methods section, in my view!
(Note that the graphical model in Fig 3C is not as helpful as having a simple equation!)

We have added the relevant equations from the methods section to Fig. 4 in the results (Line 255). As our approach to modeling task responses employs standard techniques rather than a novel contribution, we decided not to include these equations directly in the results section. However, they are now visible in the figure within the results.

3.7. 208 At the beginning of each session, we gather data from the first 33 trials, each
209 containing one data point from all unique stimuli.

How do you reward the animal during these initial 33 trials?

In the first 33 trials of each session, we rewarded the monkey based on the prior (which itself was derived from hierarchical model inference using data from previous sessions) (see Lines 305-310). We have now also included a more detailed and step-by-step explanation of how we used our method to administer rewards for our monkey in the Methods section on Lines 847-879.

3.8. 209 Using Bayesian updating, we combine the
210 behavioral choices from these trials with our prior beliefs to compute an initial estimate of the
211 posterior distribution over perceptual and decision biases. Then, we update our estimates of the
212 posterior distributions over each bias based on the animal's response in each subsequent trial.
213 This way, we can estimate the perceptual bias of the animal trial-by-trial and flexibly update
214 the reward boundary based on the estimated perceptual bias (see Methods 6.1 for more details
215 and a formal description of the model).

I appreciate that the details are in the Methods, but once again I feel it would be useful to have a little bit more detail in the main text. How do you do inference? (Are you running optimization to get a MAP estimate, or using MCMC to get samples?).

I would also expect some discussion of speed -- clearly the method needs to run fast enough to be able to compute an updated estimate of the parameters during the intertrial interval. Is your implementation fast enough for this to be the case? (How does compute time scale with the amount of past data?)

We use Gibbs sampling and then we use the mean of the posterior over the perceptual bias to compute the reward boundary (see Lines 829-845 in the Methods). Regarding computing speed, please also see our response to the reviewer's first comment (3.1.) above. Within a session, as the number of trials increased, our algorithm maintained roughly consistent online computing time (see Lines 838-845 in the Methods). Across sessions, computing time does not depend on the number of past sessions, since they are entirely summarized by the prior going into the current session.

3.9. 245 In contrast, employing a Bayesian method from an o↵-
246 the-shelf library confines one to assigning priors solely to the empirical biases. Therefore, we
247 assessed under what circumstances and to what extent our method outperforms the conventional
248 Bayesian approach, as implemented using the Psignifit library (Schutt et al., 2016).

I'm sorry, I didn't follow this. If Psignifit only allows you to infer empirical biases, how do you extract estimates of the perceptual bias?)

We thank the reviewer for raising this issue, as we had previously omitted some important details. Psignifit only allows us to infer empirical biases in all three conditions. Then, we can compute from the empirical bias estimates the underlying perceptual and decision biases if we assume the following: (1) the decision bias is well approximated by the empirical bias in the neural condition and (2) the empirical biases in the other two conditions are the sum of the perceptual and decision biases. Then, we can deconvolve the two distributions corresponding to the perceptual and decision bias random variables in the two conditions with expected perceptual biases because perceptual and decision biases are assumed to be normally distributed, independent random variables. This is now described in much greater detail in the revised manuscript (see Lines 360-367 in the Methods).

3.10. Likewise, I was puzzled by Fig 4 panels D and F. At first it looked to me like "Our Method: Psignifit: " was the title for these two plots. (It took me a while to realize these were referring to the legend for the line traces below them). But it's also unclear from the fig or caption what it means to be comparing "your method" to Psignifit. (Do you want to give your method a name?)

Thank you for the suggestion. We moved the figure legend for Psignifit and our method further away from each other in Fig.5 panels D and F (previously Fig.4). We hope they are not confusing anymore. Please also see our response to the previous comment.

3.11. Methods:

564: We used lapse rates...

This is great. I was actually thinking while reading the paper that I would like to ask you to perform some validation that the method was robust to lapses. I didn't realize until I got here that your model already incorporates lapses. So let me put in another appeal to put equation for theta_m,k into the main text. (Most readers won't bother reading the Methods section, so I think it's worth spelling out the main details of the model in the Results section!). There's no need to give all the prior distributions, but I think the basic descriptive model as well as the formulas for B_L, B_N, and B_R.

Note however that I am a bit confused because the first equation in Sec 6.1 describes the choice probabilities in terms of the cumulative normal distribution, without lapses. So is this a separate model from the one described above? Did you run some versions with lapses and others without? The two equations seem to contradict each other in the current Methods section -- please clarify!

We only used one version of the model with lapse rates. The equations in the Methods section are indeed confusing because we defined theta_m,k twice, once with lapses and once without (see Line 798 in the Methods). We corrected this in the new version of the manuscript. Please also see our response to 3.6.

3.12. Also, please number all equations!

We have now numbered all equations in the manuscript.
